# Researchers’ Experiences with IRB Oversight of Psychedelic Research

**DOI:** 10.1002/eahr.70033

**Published:** 2026-11-06

**Authors:** Logan Neitzke‐Spruill, Jill Oliver Robinson, Alison Purcell, Dominic Sisti, Amy L. McGuire, Holly Fernandez Lynch

**Affiliations:** ^1^ Postdoctoral associate at the Center for Medical Ethics and Health Policy at Baylor College of Medicine; ^2^ Research manager at the Center for Medical Ethics and Health Policy at Baylor College of Medicine; ^3^ Fourth‐year medical student at McGovern Medical School at the University of Texas Health Science Center Houston; ^4^ Associate professor of medical ethics at the Perelman School of Medicine at the University of Pennsylvania; ^5^ Leon Jaworski Professor of Biomedical Ethics and director of the the Center for Medical Ethics and Health Policy at Baylor College of Medicine; ^6^ Associate professor of medical ethics and law at the Perelman School of Medicine at the University of Pennsylvania

**Keywords:** psychedelics, institutional review board (IRB), research ethics, research oversight, investigator perspectives

## Abstract

Previous research raises concern that conservative institutional review board (IRB) approaches regarding stigmatized issues and vulnerable groups could similarly arise as an impediment to research with psychedelic drugs. We interviewed 20 US researchers who had been a principal investigator on at least one research protocol involving psychedelics about their experiences with IRB oversight and perspectives regarding what IRBs need to know. Respondents reported that their IRBs raised concern and asked questions about participant safety, consent, legal issues, and data security and confidentiality, typical domains for IRB consideration. They also mentioned several IRB knowledge gaps or misunderstandings about psychedelics, primarily about risks and safety, while emphasizing the importance of IRBs’ understanding of psychedelic drug effects. Despite IRB concerns and misunderstandings, all study respondents ultimately reported positive or neutral current experiences with IRBs reviewing their protocols for research with psychedelics, which they often attributed to cultivating strong IRB relationships and helping IRBs achieve adequate knowledge about these types of studies. Although investigators in our study did not perceive their IRBs to be a significant barrier to research with psychedelics, less experienced investigators and IRBs may face different outcomes. As research with psychedelics continues to increase, both researchers and IRBs should be responsive to continued scientific developments and emergent best practices.

Research into the clinical potential of a wide range of psychedelic drugs has generated considerable excitement about the possibility that these substances will transform individual mental health and mental health care more broadly.^1^ The excitement about psychedelic research recently culminated in an executive order issued by President Donald J. Trump, which contained several provisions to accelerate research and access to psychedelics for mental health issues. Yet, the expansion of psychedelic science faces a number of challenges, including the drugs’ current classification as Schedule I controlled substances, their shifting legal status across US jurisdictions, and the general stigmatization of psychedelics as mind‐altering drugs.^1^, ^2^, ^3^ It is possible that these challenges, and others, could lead to frustrating hurdles and protracted review processes by institutional review boards (IRBs), thereby inhibiting important scientific development.^4^

IRBs are responsible for protecting the rights, safety, and overall welfare of human research participants. Effective IRBs must be expert in both regulatory requirements and broader ethics standards for research, have adequate capacity and resources to understand the relevant science, and be aware of additional legal standards that may be relevant, such as those governing controlled substances.^5^ Ideally, IRBs are capable of both protecting research participants and facilitating important research.

If IRBs lack sufficient expertise, significant ethical problems may occur. On the one hand, IRBs may inadequately protect research participants because they do not know what to look for or what safeguards to demand, risking harm and loss of trust. On the other hand, when faced with novel research protocols, IRBs may overprotect participants due to fear of the unfamiliar and failure to recognize similarities with more traditional research, leading to inappropriate exceptionalism, unnecessary inhibition of research (including through self‐censoring by researchers), and loss of possible societal benefits.^6^ Importantly, both under‐ and overprotection can occur in reviews of the same research protocol.

Given their primary focus on participant protection, IRBs sometimes trend toward conservative oversight, especially when reviewing research on stigmatized issues or with groups perceived as vulnerable,^7^, ^8^ such as those with mental health conditions and children.^9^, ^10^, ^11^ The same is true for IRB oversight of research involving illicit substances.^12^, ^13^ IRBs are also known to be highly variable in how they evaluate risk and respond to vulnerability,^14^, ^15^ and there is substantial tension in IRB efforts that seek to protect participants by excluding key populations from research, leading to a lack of important evidence generation for those groups.^8^

Beyond these challenges, IRB oversight of psychedelic research could be further complicated by contemporary “hype” about psychedelics’ anticipated benefits, as well as insufficient knowledge about psychedelic drugs and the psychedelic‐assisted therapy model—both consequences of the widespread suspension of psychedelic research after the passage of the Controlled Substances Act of 1970. Ethical standards in several domains of psychedelic research also remain unsettled and subject to debate, such as how best to achieve informed consent in the context of research that involves altered states of consciousness and unpredictable individual experiences, as well as what protections are appropriate to address enhanced suggestibility, among other issues.^16^, ^17^, ^18^ A recent survey of IRBs suggests that IRB chairs are concerned about several issues in psychedelic research such as bias stemming from expectations of benefit, participant safety, and consent challenges.^19^ However, IRBs may be unsure how to resolve these concerns given many open questions in the field of psychedelic research.

Barriers to conducting psychedelic research have been documented in the literature.^4^ Yet little is known about how investigators conducting these studies have navigated IRB review and oversight of their research protocols. We examined these experiences, as well as researchers’ perspectives regarding what IRBs need to know to facilitate high‐quality oversight of psychedelic research.

## STUDY METHODS

We conducted interviews with individuals who have served as the principal investigator on at least one psychedelic research protocol involving human subjects in the United States, including clinical trials, neuroscientific research, and social scientific research. For the purposes of this study, we defined the term “psychedelic” to include classic psychedelics, such as psilocybin, lysergic‐acid diethylamide (LSD), or dimethyltryptamine (DMT), and substances such

**There are several features of psychedelics and psychedelic research that may pose challenges to IRBs, especially those reviewing psychedelic protocols for the first time.**

as ibogaine, 3,4‐methylenedioxymethamphetamine (MDMA; commonly known as ecstasy), or 5‐methoxy‐N,N‐dimethyltryptamine (5‐MeO‐DMT), which have distinct phenomenological and pharmacological profiles yet share properties with classic psychedelics.

Researchers were recruited using purposive sampling and identified through clinical trials listed at ClinicalTrials.gov. We contacted researchers via email with up to three additional reminders and offered a $100 online gift card for participation. Researchers who expressed interest in participating were provided a preinterview questionnaire asking about their experience as a psychedelic researcher and demographic information, such as age, gender, and race and ethnicity (see appendix A available online; see information about accessing this material in the “Supporting Information” section at the end of this article). This study was deemed exempt by the University of Pennsylvania IRB and approved by the Baylor College of Medicine IRB.

Interviews were conducted from April 2024 to November 2024 using a semistructured guide (see appendix B online) consisting of five broad domains: (1) participants’ background in psychedelic research and perceptions of psychedelic science; (2) experiences submitting protocols for research with psychedelics to IRBs, including the types of concerns raised by IRBs; (3) perceptions of IRBs’ competence in reviewing psychedelic research; (4) ethical issues related to psychedelic research; and (5) recommendations for researchers and IRBs to facilitate review of psychedelic protocols. The interview guide was piloted with two study advisors experienced in psychedelic research and IRB review processes. The guide was revised per pilot participants’ feedback to improve clarity and ensure relevance of interview questions. Interviews were recorded, professionally transcribed, and reviewed by members of the research team to remove identifying information.

Interviews were conducted until informational redundancy and thematic saturation were achieved.^20^ Interview transcripts were analyzed using thematic content analysis. A coding scheme was initially developed based on the interview guide; transcripts were then inductively coded for emergent themes by one member of the study team and iteratively refined by using a consensus coding approach with two other members. Variations in coding were discussed until consensus was achieved.

## STUDY RESULTS

We contacted 58 psychedelic researchers and conducted interviews with a total of 20. Seven individuals declined participation, and nine gave no response following our maximum number of contact attempts. Two individuals were deemed ineligible after receiving more information, and we did not follow up with 20 individuals who had been contacted at least once because we reached thematic saturation in conducted interviews. Participant characteristics are reported in table 1. Most participants were male (n = 16), white (n = 17), and older than 30 years of age (mean = 50 years).

**Table 1:** Psychedelic Investigator Sample Characteristics

***Characteristic***	*N (%)*
**Age in years (n = 19)** *	
30‐39	3 (15.8)
40‐49	8 (42.1)
50‐59	3 (15.8)
60+	5 (26.3)
Median age	44
Min. age	30
Max. age	77
**Gender (n = 19)** *	
Male	14 (73.7)
Female & other	5 (26.3)
**Race and ethnicity (n = 19)** *	
White	16 (84.2)
Hispanic	4 (21.1)
White and Hispanic	3 (15.8)
Other†	3 (15.8)
**Types of research conducted (n = 20)**	
Administration studies	20 (100)
Social science	13 (65.0)
Long‐term follow up	12 (60.0)
Other	1 (5.0)
**Types of IRB worked with (n = 19)** *	
My own university/academic medical center IRB	17 (89.5)
Another university/academic medical center IRB	6 (31.6)
Commercial/independent IRB	12 (63.2)
A central IRB (e.g., NCI CIRB)	3 (15.8)
**Years of psychedelic research experience (n = 19)** *	
1‐5 year	6 (31.6)
6‐10 years	6 (31.6)
11‐15 years	4 (21.1)
16‐20 years	1 (5.3)
20+	2 (10)
Median	8
Min.	2
Max.	36

* One participant did not answer all items in the preinterview questionnaire.

† American Indian, Native American, or Alaska Native, Asian America, and “Prefer to Self‐Describe” were collapsed due to small N's.

All 20 participants reported conducting studies involving administration of classic psychedelics or MDMA to human subjects. Thirteen participants also reported experience conducting social science research involving psychedelics using qualitative and/or quantitative methods, and 12 reported conducting long‐term follow‐up research of individuals who have used psychedelics. One researcher also reported conducting observational research of psychedelic use in retreat settings. Their median length of experience conducting psychedelic research was eight years. Nearly all (89.5%) researchers reported submitting protocols to the IRB at their own university or academic medical center, and more than half (63.2%) indicated that they have used a commercial/independent IRB.

## IRB CONCERNS ABOUT PSYCHEDELIC RESEARCH

Researchers reported a wide variety of specific concerns raised by IRBs about their psychedelic research protocols. In general, however, the types of concerns they reported suggest that the IRBs they interacted with did not treat psychedelic research exceptionally. Specifically, the concerns raised often related to participant safety, consent, legal concerns, and data security and confidentiality, with the latter arising particularly in relation to social science protocols in which participants may report prior illicit use of psychedelics. Some described the concerns that IRBs raised as unsurprising, given their responsibilities. One researcher stated, “I think, as a whole, [the concerns raised are] largely around things that should be expected in terms of the scope of practice for an IRB. So it's things around consent content, interaction with participants, recruitment, … [and] the ethics around people having access to getting into the study and their ability to understand the risks associated with participating” (Participant 105). Another researcher explained: “I haven't had any questions come back that just seemed ridiculously ignorant. I feel like they do their homework…. They don't really care about the [research] question. They're like, ‘Are you doing it in a way that is safe?’ And I think that's their main concern. They don't really care about what your scientific objective is” (Participant 111).

Most researchers reported encountering concerns about participant safety (n = 11), including having safety precautions and emergency procedures in place during dosing sessions, adopting procedures to determine fitness for participant discharge post‐dosing, monitoring participants for physiological and psychological adverse events, and making certain that drug products are securely stored. In response to a question about what concerns the IRB raised, one researcher listed a series of methodological and scientific questions related to participant safety: “What's an appropriate dose range, and can you justify that? What are the appropriate rescue medications for a given drug administration session, and what's the protocol with which you would decide whether to administer those rescue medications or not? What are your parameters for monitoring vital signs during the drug session and how you'll respond to blood pressure excursion? Are you inquiring about suicidality, suicidal ideation behavior? And if so, what is your protocolized response if and when a person presents with some suicidal ideation or behavior that is concerning? And how are you determining psychiatric fitness for a given study, especially in patient populations, and what is a real reasonable control condition?” (Participant 104).

While researchers generally agreed that participant safety is of utmost importance, there were a few examples of IRBs insisting on safety protocols for psychedelic research that the researcher disagreed with, creating conflict. For example, one researcher (Participant 101) experienced challenges tracking participants across surveys due to his IRB's insistence on deidentifying and delinking datasets, which he attributed to the fact that questions about psychedelics were a component of the study. Another expressed frustration with IRBs that “overstepped” by insisting that participants dosed with psychedelics must be kept overnight, which this researcher and several others (n = 6 total) viewed as unnecessary: “When my teeth have gnashed the loudest is when we've had that kind of conflict with an ethics committee where they've overstepped the mark. They've said, ‘Oh, well you must keep the patient,’ when we know that A, it's not necessary, and B, the patients wouldn't want it. Would you stay in a hospital overnight if you didn't have to? It's noisy, it's unpleasant, it's not your bed. Yet an ethics committee decides that's the best for you, that drives me crazy” (Participant 120).

IRBs sometimes raised concerns about informed consent (n = 6), including issues related to who should procure it, what needs to be included in consent documents, whether consent is possible for psychedelic research, and how to effectively achieve it for studies involving psychedelic administration. One researcher described their IRB's concerns about consent as largely substantive, indicating that their interaction with the IRB was “reasonable”: “I think that [the IRB] asked legitimate questions about how can you actually get consent, what are you going to go through to help people understand what this is like? And then also understood that it is difficult to consent people for something that until you've experienced it, you can't really understand. But that's also not uncommon in some ways—the fact that this is affecting your perception so much, but you can't explain what it's like to go through chemotherapy until you have chemotherapy” (Participant 110).

Another researcher noted that their IRB was primarily concerned with *who* obtained informed consent: “There was also, around the consent process, somewhat of a tedious situation in which we were required to have the psychiatrist do all of our consents, which is very expensive and just hard to get that time from psychiatrists” (Participant 115).

This researcher went on to specify that the additional expense associated with requiring psychiatrists to procure informed consent was often wasted because the initial screening process “ends up screening out about 50% of the people for our clinical trials” (Participant 115). Other researchers mentioned that IRBs focused on the language in the consent document, to ensure it was accessible and that the risks were adequately described, as is typical in IRB review of research generally.

Five researchers independently reported that their IRB raised legal concerns when reviewing their psychedelic research protocols, while two others indicated that legal issues came up during the review process but were handled by an office or committee within their university other than the IRB. When asked directly, 13 researchers reported that their IRBs never raised any specific legal concerns about their research, with one noting that their IRB was not concerned with legal issues and simply deferred to adherence to US Food and Drug Administration (FDA) and Drug Enforcement Administration (DEA) guidelines as a measure of legality.

When IRBs did raise legal concerns, the most common issues pertained to handling controlled substances and questions surrounding universities’ legal liability for possible harms associated with psychedelic research. One researcher stated, “I think the other piece again has been not so much the safety of the patients, but I suppose the safety of the institution from a liability perspective of just how do we make sure that the drug remains safe and that people are held accountable for making sure that there's no issues with diversion or theft or these sorts of things?” (Participant 103). Another researcher said with regard to whether their IRB raised legal concerns, “Only indirect legal concerns. In other words, what if a participant is enraged? What if a participant runs out of the treatment center? So, you could imagine that those are in essence legal questions. I mean, they're also health and safety questions, but given the nature of an IRB, I could imagine some of it's legal, too. Like, ‘Are we responsible if someone jumps out a window? Are we responsible if a night attendant is taken hostage?’” (Participant 102).

Other legal concerns related to video recording sessions, methods of contacting participants during the study, and data sharing. These concerns sometimes led to additional administrative burdens and delays, adding to general frustration with the efficiency of the review process.

Concerns about data security and confidentiality were sometimes raised by IRBs reviewing psychedelic social science research, typically tied to concerns about stigma, legal risks of being identified as a psychedelic user, or legal risks associated with data management. Researchers’ perceptions of this type of concern varied. For instance, one felt that it was justified, noting that the IRB paid “healthy attention to issues regarding confidentiality” (Participant 119) when reviewing observational studies involving psychedelics. However, another researcher (Participant 105) felt that the data security measures requested by their IRB for social science research were “overkill.”

## IRB UNDERSTANDING OF PSYCHEDELIC RESEARCH

While researchers reported that their IRBs generally raised concerns that were reasonable and within their typical purview, they also sometimes indicated that IRBs lacked relevant knowledge or had some degree of misunderstanding about psychedelics or psychedelic research. Despite these gaps or misunderstandings, however, researchers did not report a compromised review process.

When asked to describe how well IRBs seemed to understand their psychedelic research, about half of the researchers who commented on the issue (n = 8) reported that their IRBs had adequate understanding. Several of these researchers held positions at medical schools with IRBs highly experienced with clinical research involving novel interventions and approaches, and they attributed their IRBs’ understanding to prior review experience, their own effectiveness in protocol development, and local cultures more open to and aware of psychedelics. Nonetheless, four of those who reported adequate IRB understanding still noted relevant knowledge gaps about specific aspects of psychedelics or psychedelic research. These gaps included the concept of “set and setting”—which refers to psychosocial factors that shape psychedelic experiences, i.e., the person's intentions, beliefs, expectations, mood, and personality (set) and the physical and social context in which they are having the experience (setting)^21^, ^22^—and the potential for psychedelic experiences to instigate false beliefs.

The other half (n = 9) reported that their IRB did not adequately understand psychedelics or important considerations related to psychedelic research. One researcher (Participant 115) noted that their IRB explicitly stated they did not have sufficient understanding of psychedelic research to evaluate the protocol and requested more information: “I submitted our IRB proposal to the IRB. They sent it back saying, ‘We don't have enough information to know about these studies. We need more information.’ … that's exactly what they said. Yeah, that they didn't have enough information or knowledge base to be able to accurately judge the safety of what we or any of these trials are proposing. So provide more information about what's done in the literature and what has been done” (Participant 115).

Ultimately, however, even these researchers often reported that their IRBs possessed enough general knowledge about research ethics and a willingness to work with researchers that this lack of domain‐specific knowledge did not undermine the review process. For instance, when asked whether the IRB generally understood their research, one researcher (Participant 105) who was the first to study psychedelics at their institution said that “as a whole” the IRB did not understand, yet they described the review process positively due to the support from and accessibility of the IRB chair. Several researchers explained that they had to educate their IRBs, which facilitated review of their protocols. As one researcher said, “it's been an educational course for [my IRB chair], but she's been willing to figure that stuff out, and at the end of the day, she really hasn't made it impossible” (Participant 108).

Researchers mentioned several specific points of IRB misunderstanding, including about the safety of psychedelics. For instance, one mentioned how IRBs held misconceptions about psychedelics’ risks when he submitted his first psychedelic protocol several years earlier, before psychedelics had become a more popular subject of study: “I believe there were a fair amount of misconceptions about what psychedelics do and the true risk of psychedelics. There were sort of false beliefs of how damaging this could be to patients in general. And so much of what we had to do was to ensure … safety. Although this is research that had been done very extensively 30, 40 years before. So people were asking [for] pharmacokinetic information and it's like, we don't need that. It is well established” (Participant 116).

Two other researchers working with MDMA perceived that their IRBs had misconceptions about the association between MDMA use and neurotoxicity, which they worked to dispel during the early years of the resurgence in psychedelic research. Sometimes, IRBs’ misconceptions about safety contributed to perceived misunderstandings about necessary study procedures, including experimental design and security protocols. In a more recent example, a researcher who was the first to conduct psychedelic research at their university noted a question they received about vetting participants to avoid drug diversion: “Because the IRB asked, … ‘Do we need to be considering background checks, any of these things?’ Absolutely not. That's ludicrous…. So I think just the misconceptions around what these are, how safe they are, that while, yes, I'm sure there is some risk someone is going to try to rush us in the office and break open the safe, the chances of that are so ridiculously small that we don't really need to waste too much breath thinking about it” (Participant 103).

A few researchers also reported that their IRBs either did not understand or were unaware of the importance of specific issues relating to set and setting and the importance of facilitator training. For example, a researcher who was among the first to conduct psychedelic research at his university noted it was a long process to bring his IRB up to speed on set and setting: “In my experience, it hasn't been that they have wrong knowledge, it's just that they have big gaps in knowledge. So the notion of the set and setting, and how and why there's a special room for this, and why there's other people in the room, and why we're not just giving people the psychedelics in a hospital room, I think has been a long road to get people up to speed on” (Participant 101).

Ultimately, however, this researcher was able to address the IRB's concerns. Overall, researchers reported that they were able to educate their IRBs about key concepts and issues to help advance protocol review and eventual approval.

## Psychedelic Researchers’ Overall Experience with IRBs

Despite some of the IRB concerns and misunderstandings about psychedelic research noted by researchers in our study, all 20 reported generally positive or neutral current experiences with protocol review. One researcher (Participant 106) noted their experience was “excellent” and occurred without “any obstacles or barriers” during the IRB review process. Although two researchers recounted examples of negative experiences with their IRBs, these were for research protocols submitted during the early years of the contemporary psychedelic science movement. One researcher with more than 20 years of experience studying psychedelics explained that the process has “evolved over time”: “[My first protocol] was quickly rejected, probably unanimously. I think the only comment I ever heard was, ‘What, are you kidding me? You can't study this.’ … After that, I had no IRB problems to speak of” (Participant 109).

In more recent years, researchers described the review process as less challenging than they anticipated. A few participants noted surprise that their IRB did not push back more. One researcher said, “Yeah, so the IRB was not as challenging as I thought it was going to be. I thought we would get a lot of pushback from the IRB. We got a lot of pushback from [an oncology‐specific review board] but the IRB process was much less challenging … ” (Participant 110).

Others lauded their IRBs for adopting a value‐neutral, “principled” approach that resisted psychedelic exceptionalism, treating psychedelic research like any other research topic. As one researcher noted: “I never felt there [was] apprehension in approving a psychedelic study because of psychedelics, because of the stigma around psychedelics. I felt their openness and they were judging psychedelic studies as they would judge any other study based on the quality and the clarity of the protocol and the consent and the fact that people's safety is central to how the study is designed and that the consent is accurate and so on” (Participant 118).

Several researchers highlighted the importance of IRB experience with and knowledge about psychedelic research, as well as researchers’ ability to work collaboratively with the IRB to build rapport, provide education, receive feedback prior to submission, and address concerns. One researcher said: “Thankfully, I work in a small, discreet environment in which the chair of my IRB is four doors down the hall from my office, and if she's got questions, she asks me; if I got questions, I ask her. And so we're able to come up with something both of us can live with” (Participant 108). Another researcher explained, “In general, my experience with all of them has been positive, and that doesn't mean there hasn't been challenges along the way …. I have personally experienced that the IRB is my friend. I sometimes need to educate my friend on things that they don't understand about my work, but they have been open to me doing that…. And I think the most important thing that I've experienced here especially, is the chair overseeing all of the IRBs is incredibly accessible and supportive and has really helped to facilitate those conversations” (Participant 105).

Although most of the researchers in our study expressed positive or neutral sentiments regarding their experiences with IRB review of psychedelic protocols, several (n = 8) also noted that their psychedelic protocols received greater scrutiny relative to other types of research. Some researchers attributed this to the nature of the topic or the lack of appropriate knowledge about psychedelic‐assisted therapy. In contrast, some linked heightened IRB scrutiny to increased knowledge about psychedelic research, which provided a basis for the IRB to ask more detailed questions (e.g., about the credentials of facilitators or specific procedures associated with dosing sessions). While some considered heightened scrutiny of their psychedelic protocols “paternalistic” and unwarranted, others did not recount it as a major hindrance despite the additional time and effort.

## ESSENTIAL KNOWLEDGE FOR IRBs REVIEWING PSYCHEDELIC RESEARCH

When asked what IRBs need to know or be told about psychedelics to adequately review protocols, researchers most often suggested that IRBs be familiar with psychedelics’ drug effects (n = 7; see table 2). This often included knowing about the risks and safety profile of psychedelic drugs in general, the differences between the various substances referred to as psychedelics, and the differences between psychedelics and other drugs. For example, researchers said that IRBs should be aware of cardiovascular contraindications, vulnerability associated with the acute effects or processes of psychedelic‐assisted therapy, and the potential for psychological destabilization. According to one researcher: “They need to know differences between psychedelics and different compounds, that they're not all the same and created equal in terms of their impact on one's mental status, and also in terms of the physiological and cardio toxicity, there's differences there…. They need to understand that these can induce experiences that persist after the dose, which is what we want to harness clinically, but that can often be destabilizing” (Participant 106).

**Table 2:** Essential Knowledge for IRBs Reviewing Psychedelic Research

What investigators think IRBs should know about psychedelics	n (%)
Psychedelic drug effects	7 (35)
Risks	6 (30)
Safety profile	5 (25)
Set and setting	4 (20)
Long‐term effects	3 (15)
Differences between drugs	2 (10)
Issues surrounding definition and reporting of adverse events	3 (15)
Consent issues	3 (15)
Expectation effects	2 (10)
Participant support	2 (10)
Historical precedent for psychedelic use	2 (10)
Diverse populations	2 (10)
Treatment procedures	1 (5)
Touch	1(5)
Other	7 (35)

One researcher who had experience both submitting psychedelic research protocols and working as a member of the IRB suggested that IRBs should be aware of the full extent of the risks posed by psychedelics’ effects: “A well‐formed protocol should really review the medical and psychiatric risks of psychedelics … including facts such as any estimated LD50 [median lethal dose] of psilocybin, for instance, is likely very many thousands of times larger than what is currently considered the therapeutic dose…. But that also the main risks of psychedelics are psychiatric in nature, that people during the acute drug effects of a therapeutic dose, of [a] high therapeutic dose of psilocybin especially, can really be incredibly vulnerable and can experience truly the entire range of human emotions” (Participant 104).

For this researcher, IRBs need to understand the risks of psychedelics as well as the likelihood of those risks, which can help convey the relative safety of working with psychedelics. This researcher went on to state that it is important for investigators to make clear to IRBs that difficult drug experiences should not necessarily be considered risks or a negative outcome. Other researchers also asserted that psychedelics are relatively safe overall, especially in controlled settings. Researchers viewed this as essential knowledge for IRBs, as reflected by one researcher's comment: “I think they just need to have a fair and balanced understanding of the physiological, biological, and psychological effects of the drugs. And from a physiological standpoint, there's very low risk there. Biologically, the class of psychedelics in particular are quite benign. And then psychologically is where you see the majority of the quote unquote risks. And if you have the right screening and preparation in place, then those risks are very well mitigated” (Participant 107).

A few researchers also emphasized that IRBs should be aware of the potential for long‐term effects of psychedelics. As one researcher explained: “I think there could be a little more scrutiny around the long‐term effects of psychedelics that extend beyond termination from the study. At the end of the day, if they're terminated, they're not within the IRB purview anymore. We try to prep our participants and part of that is informed consent to understand that the duration of the effects, you may find changes that are positive in one or many domains, but because of the disruptive nature of the psychedelic and how quantum and acute changes can occur, that that can destabilize other aspects of their life, sometimes for good later on, but other times it can just be more challenging. I think if IRBs knew that element, that would be important—I mean really understood that piece” (Participant 106). Relatedly, a few researchers mentioned the importance of having IRBs understand the impact of set, setting, and “the environment in general” on psychedelics’ effects.

Some researchers mentioned an array of other specifics that IRBs should know about, such as the need for psychological support, difficulties associated with achieving informed consent for psychedelic experiences, the importance of diverse representation in trials, and the historical and cultural precedent for psychedelic research. Yet a few researchers noted that IRBs do not need any “specific knowledge” about psychedelics or anything beyond what is ordinarily required for evaluating clinical research protocols. Several investigators implied the point that some (n = 5) explicitly mentioned, that educating the IRB about the state of psychedelic knowledge and areas of uncertainty is the responsibility of investigators, especially because many IRBs are not used to reviewing psychedelic protocols. One researcher stated, “Even in the field, there is a lot of uncertainty or discussions around what should we call adverse events and how should we monitor for adverse events in psychedelic research, right? It's an uncharted territory…. So is the IRB aware of that and looking into that and have expectations for researchers to mention this in the informed consent and include this in their safety protocols in the protocol? I don't know. I don't think so. I think they're learning from us. At least in my IRB, I think I've taught them what psychedelics are and how to think about safety and consent. So I think part of this responsibility is very much on the investigator's side right now” (Participant 118).

As a solution to these dilemmas, which are to some extent inherent to the IRB's task of reviewing diverse research protocols, one researcher (Participant 114) suggested that “knowing when to call in an outside expert” is essential knowledge for IRBs tasked with reviewing psychedelic research.

## DISCUSSION

This qualitative study with researchers who conduct studies with psychedelics sheds light on their interactions with the IRBs responsible for overseeing this growing field of research. Despite encountering an array of concerns and questions about their studies, researchers generally reported positive experiences with their IRBs overall. IRBs predominantly raised concerns about safety, other participant protections, and consent, which researchers often viewed as reasonable and within the appropriate purview of the IRB. Although researchers encountered some misunderstandings and gaps in IRB knowledge about psychedelics, they were able to overcome these challenges by providing information and education to their IRBs. Overall, researchers did not perceive their IRBs to be a significant barrier to psychedelic research, in contrast to some of the literature that motivated this study in which IRBs were reported to be conservative in the context of mental health research, research involving illicit substances, and research with vulnerable populations.^9^, ^10^, ^11^, ^12^, ^13^ Notably, this finding aligns with a recent survey of IRB chairs, which concluded that they are generally open‐minded about psychedelics, with reasonable concerns given the nature of their oversight and evolving nature of psychedelic research as a field.^19^

Considering research generally, complaints from researchers about IRBs are common.^23^, ^24^, ^25^, ^26^ For example, Lynch et al. note that researchers point to “the level of work required to comply with IRB requests, a perceived lack of scientific expertise among reviewers, nit‐picking requests for changes, excessive attention to limiting institutional risk, and stifling innovation, … [while] [e]mpirical studies have found evidence of IRB delay, inconsistency, mission creep, opacity, conservativism, reputational protection, emphasis on form over substance, and discomfort with risk‐benefit analysis.”^27^ Although these are serious concerns and there have been numerous efforts to improve IRB quality overall,^28^ part of the challenge facing IRBs is that they are intentionally placed as gatekeepers between researchers and researchers’ ability to conduct unfettered research with human subjects. As a result, IRBs’ protocol review and questions about a research study inevitably slow research compared to the absence of IRB oversight but with the essential purpose of providing independent review focused on ensuring adequate participant protection. Yet the essential purpose of the IRB review process is to provide independent review of research and to ensure there is adequate participant protection. The goal is for IRBs to ensure that protection without unduly impeding ethical research, working in close collaboration with researchers.

Although it may not always work as intended, this collaborative relationship is critical because IRBs are often generalist bodies, responsible for reviewing a wide variety of studies. To the extent that IRBs have specialized knowledge, it is typically only in the sense of reviewing biomedical research versus social science research, with additional subject matter specialization to focus on cancer protocols or pediatric research in some cases. Larger institutions and commercial IRBs with multiple boards or panels may further assign protocols for review by disease area or other categorization, although these groupings may differ across IRBs and the research sites. Regardless of these efforts to build and take advantage of member expertise, IRBs cannot reasonably be expected to have independent expertise in *all* types of science, interventions, methodologies, or specific protocols that may come before them, especially in emerging and complex areas. Indeed, even the US regulations governing research with humans expect relatively general expertise, calling for IRB members to have “varying backgrounds to promote complete and adequate review of research activities commonly conducted by the institution” and for IRBs to be “sufficiently qualified through the experience and expertise of its members … to promote respect for its advice and counsel in safeguarding the rights and welfare of human subjects.”^29^ The regulations go on to state that IRBs must include members knowledgeable regarding institutional policy, applicable legal requirements, and standards of professional conduct. Importantly, to fill relevant gaps—especially as they might arise on a protocol‐by‐protocol basis, IRBs are permitted to “invite individuals with competence in special areas to assist in the review of issues that require expertise beyond or in addition to that available on the IRB,”^29^ typically referred to as “outside experts.”

Ultimately, IRBs at least must have sufficient expertise to apply relevant research ethics and regulatory frameworks to protocols, which entails being able to determine which questions to ask researchers and when they should seek outside expertise or turn to the literature themselves for additional independent input.^6^, ^30^, ^31^ Although IRBs should be wary of relying exclusively on researchers to provide all relevant information needed for protocol assessment, given investigators’ inherent interest in allowing their research to proceed and likely perspective that their protocols are acceptable as submitted, the research team will—in many cases—be far more expert in the area of their science than the IRB. Thus, it is reasonable to expect researchers to bear a substantial responsibility to bring their IRBs up to speed on the science and relevant standards in the field, as needed, especially at institutions where psychedelic research has not been previously conducted.

Experienced researchers in our sample clearly recognized this obligation and sought to meet it regarding their psychedelic research protocols, likely contributing to their generally positive experiences. Newer psychedelic researchers should learn from this finding. IRB chairs noted in a recent survey that they could use further education and resources regarding how best to protect participant safety in psychedelic research and ensure appropriate consent.^19^ Similarly, researchers we interviewed noted a range of information that, if better understood by IRBs, could help alleviate concerns and time‐consuming exchanges stemming from unfamiliarity with psychedelics or procedures for psychedelic‐assisted therapy. Researchers emphasized that IRBs need a thorough understanding of psychedelic drug effects, including the range of subjective effects psychedelics can elicit, anticipated risks, as well as the existence of an overall favorable safety profile, especially for participants without contraindications. Another area that could be especially important to clarify for IRBs without substantial prior experience reviewing protocols for psychedelic research is the current debate regarding what should be considered an “adverse event” in the context of psychedelic research, given that certain subjective phenomena may be expected or even associated with therapeutic outcomes, including euphoria or frightening visualizations.^32^, ^33^ At present, many IRB chairs have acknowledged gaps in their understanding of psychedelic risks and benefits,^19^ which, if addressed by researchers, could help avoid hesitancy or excessive scrutiny by IRBs not steeped in psychedelic science. Ultimately, more knowledgeable IRBs will be better equipped to identify and anticipate issues with protocols to avoid safety risks and better protect participants, without unreasonably inhibiting the science.

Building IRB expertise and awareness regarding psychedelic research will be increasingly important as more researchers who are themselves less experienced working with psychedelics begin submitting protocols as interest continues to grow and psychedelic research is normalized.^3^, ^34^, ^35^ In these settings, IRBs should be especially encouraged to call on outside experts absent strong psychedelic knowledge and experience among existing board members. This will also likely be helpful while major debates in psychedelic research remain unresolved, such as whether psychological support or post‐dosing integration sessions are essential for safety and efficacy,^36^, ^37^, ^38^ or how to promote adequate informed consent to experiences that may lead to fundamental transformations to personality, values, or beliefs.^18^, ^39^, ^40^, ^41^, ^42^, ^43^ Where there is no single right answer, experts can provide independence from researchers and help IRBs weigh relevant considerations.

Researchers in our study who reported positive experiences with their IRBs often credited their protocol design and the rapport they had built with IRB members. Thus, one way to improve the IRB review process for psychedelic research is by spending adequate time prior to submission to produce an effective protocol that provides comprehensive background information on the relevant science, risks and benefits, ethical considerations, and justifications for the procedures involved, while cultivating relationships with the IRB to build trust and open communication. This recommendation applies to all types of research but especially to novel topics an IRB may be encountering for the first time. Consulting with IRB members to explain research plans and anticipate concerns prior to submission can also improve review efficiency and outcomes.

Despite researchers in our study describing overall positive experiences with IRBs, some nonetheless reported that their psychedelic research protocols received heightened scrutiny compared to other types of research, in some cases leading to a challenging review process. It is unclear, however, whether heightened scrutiny reflects overprotection in psychedelic research or instead indicates appropriate oversight. As the evidence base and ethical standards for psychedelic research undergo continued refinement,^44^, ^45^ it will be essential for IRBs to avoid undue conservativism. Importantly, however, our interviews with psychedelic researchers, alongside a recent survey of IRB chairs, suggest that IRBs are asking appropriate questions to advance their participant protection obligations without unduly impeding psychedelic research, a welcome change from concerns about conservative approaches identified in other areas of research.^8^, ^9^, ^10^, ^11^, ^13^, ^19^ Further research will be essential to determine whether these findings extend beyond experienced psychedelic investigators and IRBs.

## STUDY LIMITATIONS

Our study sample was made up of experienced psychedelic researchers who, as a condition of inclusion, had achieved success obtaining IRB approval for psychedelic research protocols. This is an important limitation, as it likely skews our results toward more positive perceptions of IRB oversight. It is possible that researchers new to conducting psychedelic research or researchers at different institutions where psychedelic research protocols have never been reviewed would report more significant challenges receiving IRB approval—and that researchers who had not successfully attained IRB approval of a psychedelic research protocol would offer evidence of conservative, overprotective approaches generally missing from our sample. However, even highly productive researchers who have conducted a lot of psychedelic studies still reported some concerns. Our sample was also predominantly made up of researchers who had conducted clinical trials, which may have shaped the nature and type of concerns they encountered during the IRB review process. IRBs that focus on protocols involving social science studies may be more sensitive to issues such as confidentiality, as opposed to safety. Finally, researchers reported IRB concerns and knowledge they perceived essential for IRB review in an open‐ended manner. We did not survey each researcher about each concern or aspect of essential knowledge, such that failure of any researcher to raise that issue cannot be interpreted as evidence of its absence.

## CONCLUSION

An increasing number of researchers are likely to submit psychedelic research protocols to IRBs as science advances and cultural attitudes continue to shift toward greater acceptance and even hype regarding psychedelics. Our findings suggest that experienced IRBs are unlikely to pose an unreasonable barrier to psychedelic research or miss important aspects relevant to participant protection. However, there are several features of psychedelics and psychedelic research that may pose challenges to IRBs, especially those reviewing psychedelic protocols for the first time. In the event that IRBs lack specific subject matter expertise about psychedelics, researchers conducting psychedelic studies are well‐equipped to inform IRBs about essential scientific and ethical considerations by building trust and rapport with IRB members, crafting clear and comprehensive protocols, and serving as IRB members or outside experts. Both researchers and IRBs should be responsive to continuing scientific developments and best practices for psychedelic research, while remaining open to measured, safe, and ethical experimental progress.

## ACKNOWLEDGMENT

This research was funded by The Greenwall Foundation.

## DISCLOSURE

HFL reports unrelated research funding from Arnold Ventures, the National Institutes of Health, and ARPA‐H; paid travel to deliver a lecture at WCG's annual IRB training; and paid travel and honorarium to deliver a lecture to Eli Lilly on FDA approval standards. She is an emeritus member of the Board of Directors of Public Responsibility in Medicine & Research (PRIM&R), co‐chair of the Consortium to Advance Effective Research Ethics Oversight (AEREO), and advisory council member to the OPEN Foundation's Advanced Education in Psychedelic Therapy Training Program (ADEPT).

ALM reports unrelated research funding from the Ortus Foundation, the Hollie Stenson Foundation, Arnold Ventures, National Endowment for the Humanities, and the National Institutes of Health. She is a paid member of the scientific advisory board for Geisinger Research and Morgridge Institute for Research and is on the board of directors for the Greenwall Foundation. She did not receive any support from the Greenwall Foundation for her work on this project.

## Supporting information

The appendices are available in the “Supporting Information” section for the online version of this article and via *Ethics & Human Research*'s “Supporting Information” page: https://www.thehastingscenter.org/supporting‐information‐ehr/.

Supporting Information
