# Supplementary material for: Researchers’ Experiences with IRB Oversight of Psychedelic Research
Source: Ethics Hum Res. 2026 Nov 6;48(6):6–19. doi: 10.1002/eahr.70033 (PMC13743504; doi:10.1002/eahr.70033)
Supplement: Supplementary file 1 [file EAHR-48-6-s001.pdf]

Researchers' Experiences with IRB Oversight of Psychedelic Research

LOGAN NEITZKE-SPRUILL, JILL OLIVER ROBINSON, ALISON PURCELL, DOMINIC SISTI,
AMY L. MCGUIRE, AND HOLLY FERNANDEZ LYNCH

APPENDICES

Investigators' Experiences with IRB Oversight of Psychedelic Research

Appendix A: Pre-Interview Questionnaire

Pre-Interview Questionnaire

Thank you for agreeing to participate in our interview study. Before the interview, it would be helpful to have some background about you and your experience with psychedelic research. Your responses to this questionnaire will be treated confidentially and will not be reported at the individual level. You may skip any questions you prefer not to answer.

1. What is your name.

2. Please list your degrees and relevant disciplines.

3. Approximately what year did you begin conducting psychedelic research protocols involving human participants?

4. Which psychedelic substances have you studied in research involving human participants and, if applicable, for which indications?

5. Which of the following types of psychedelic research do you conduct? Select all that apply.

    - ☐ Administration of psychedelics to human participants
      [If yes selected]
        - For how many research protocols involving administration of psychedelics to human participants have you served as the principal investigator? For how many research protocols involving administration of psychedelics to human participants have you served as a co-investigator?
    - ☐ Social science, e.g., interviews and surveys regarding psychedelics
      [If yes selected]
        - For how many social science psychedelic research protocols involving human participants have you served as the principal investigator?
        - For how many social science psychedelic research protocols involving human participants have you served as a co-investigator?

- ☐ Long-term follow up of individuals who have psychedelic experience
  [If yes selected]
    - o For how many research protocols involving long-term follow-up of psychedelic use have you served as the principal investigator?
- ☐ For how many research protocols involving long-term follow-up of psychedelic use have you served as a co-investigator? Other, please describe:
  [If yes selected]
    - o For how many of these other types of psychedelic studies have you served as the principal investigator?
    - o For how many of these other types of psychedelic studies have you served as co-investigator?

6. Thinking about all psychedelic research where you have been the principal investigator or a co-investigator which types of IRBs have been the IRB of record responsible for the ethical review of the research? ? Select all that apply.
   - ☐ My own organization's University/academic medical center IRB
   - ☐ Another University/academic medical center IRB
   - ☐ Commercial/Independent IRB
     A central IRB like NCI CIRB
   - ☐ Other, please describe:

7. About how many non-psychedelic research protocols involving human participants have you been involved in?
   9a. Of these non-psychedelic research protocols, how many have you served as the principal investigator?
8. Have you worked with psychedelic research organizations outside your primary work place?
   - ☐ Yes
   - ☐ No

   If yes, please list which organizations and for how long: _______________

9. Have you ever served on an IRB?

   - ☐ Yes
   - ☐ No

   If so, when and in what capacity (e.g., chair, member)?

10. Where do you currently reside? (city, state, country)

11. What is your current age in years?

12. What is your gender?
    - ☐ Female
    - ☐ Male
    - ☐ Non-binary/genderqueer
    - ☐ I prefer to self-describe or none of these options describe me: (please describe)
    - ☐ Prefer not to say

13. Which category or categories best describe you? Select all that apply.
    - ▢ American Indian, Native American, or Alaska Native
    - ▢ Asian or Asian American
    - ▢ Black or African American
    - ▢ Hispanic or Latino
    - ▢ Native Hawaiian or Pacific Islander
    - ▢ White or European American
    - ▢ Middle Eastern or North African/Mediterranean
    - ▢ I prefer to self-describe/none of these options describe me, please describe:
    - ▢ Prefer not to say

Appendix B: Interview Guide

Background

Let's start with some background about your psychedelic research:

1. How would you describe the main research questions driving your work?
2. What is the one thing you are most excited about regarding the current state of psychedelics and psychedelic research? What is the one thing you are most concerned about? [Pilot this question for placement; should it go at start or end of the interview; don't let this go too long.]

Experience with IRBs

Now I'd like to hear more about your experience interacting with IRBs reviewing and overseeing your psychedelic research.

3. How has that gone, overall?
    - Has the experience gone as you predicted it would?
    - Has anything been surprising?
4. Have you worked exclusively with your institution's IRB in reviewing your psychedelic protocol(s) or also with IRBs at other institutions and/or commercial IRBs (e.g., WCG, Advarra)?
    - Within your institution, has your psychedelic research been reviewed by an IRB focused only on biomedical research or social science research?
    - If you have experience across multiple IRBs reviewing psychedelic research, how do they compare? *(And if so, probe for differences across the questions below, too.)*
5. Do you think the IRB(s) responsible for reviewing your psychedelic protocol(s) understood your research?
    - What, if anything, did they not understand?
    - What did you do to improve understanding, if needed? Were you successful?
6. What, if any, methodologic or scientific concerns have IRBs raised about your psychedelic research?
    - Did you agree with these concerns?
    - How did you resolve those concerns?
    - Were you satisfied with the resolution?
    - *If relevant:* How did these concerns differ across IRB type or type of study protocol?
    - *If ever conducted administration study*: Has an IRB ever asked questions or raised concerns about the clinical space (i.e. setting) where psychedelics would be administered?
7. What, if any, legal concerns have IRBs raised about your psychedelic research?
    - Did you agree with these concerns?

- How did you resolve those concerns?
- Were you satisfied with the resolution?
- *If relevant:* How did these concerns differ across IRB type or type of study protocol?
- *If ever conducted administration study*: What, if any, specific concerns have IRBs raised about DEA, FDA, or other regulatory requirements associated with your psychedelic research?

8. *If ever conducted administration study*: Have you experienced any tension between IND requirements or guidance from the FDA and your IRB?
9. What, if any, ethical concerns have IRBs raised about your psychedelic research?
    - Did you agree with these concerns?
    - How did you resolve those concerns?
    - Were you satisfied with the resolution?
    - *If relevant:* How did these concerns differ across IRB type or type of study protocol?
10. Has an IRB ever identified an important ethical consideration about your psychedelic research that you hadn't thought about before or that made you think differently?
    - If so, what was it? How did you respond?
11. Has an IRB ever missed what you thought was an important ethical consideration in your psychedelic research?
    - If so, what was it? How did you respond?
12. How, if at all, have your interactions with IRBs in the context of your psychedelic research differed compared to other types of studies you've been involved with?
    - Do you think IRBs treat psychedelic research differently?
    - Why/how? Should they?
13. Is there any psychedelic study you think would be important to conduct but you anticipate an IRB would not allow?
    - What was the issue and what made you think they would not allow it?
    - Did you discuss the anticipated concerns with the IRB? Why/why not?

Perceptions of IRB Competence

Now I'd like to ask about your general thoughts on IRBs reviewing psychedelic research.

14. Do you think IRBs do a good job reviewing psychedelic research? Why/why not?
15. What are their strengths in this area? What are their weaknesses?
    - Is there anything would you like to see IRBs do differently in reviewing psychedelic research?
16. In general, do you think IRBs have any misconceptions about psychedelics and/or psychedelic research?
    - If so, what are they?
    - What could help overcome those misconceptions?
17. What do you think IRBs need to understand about the science to competently review psychedelic research?

- Do you think they understand that now?
- What resources could help them? What's standing in the way?

Ethical Issues

18. What do you think are the most significant ethical issues arising in psychedelic research that IRBs need to be aware of?
    - Do you think they are aware of those issues now?
    - What resources could help them? What's standing in the way?
19. In general, what risks should IRBs be looking for when reviewing psychedelic research?
    - What concerns, if any, do you have about social or legal risks to participants in psychedelic research? How should those be addressed?
    - What concerns, if any, do you have about the privacy of participants in psychedelic research?
20. In general, what protections should IRBs require for participants in psychedelic studies?
    - What protections do you think go too far? Why?
21. In general, how should IRBs think about the potential benefits of psychedelic research?
    - What role, if any, can IRBs play in mitigating hype about those benefits?
22. What is the ideal approach to consent for psychedelic research?
    - In general, what's essential for participants in psychedelics research to know?
23. How should physical touch between facilitators and participants should be handled in psychedelic research?
24. Which populations, if any, should generally be excluded from psychedelic research? Why?
    - Which populations, if any, are at risk of being inappropriately excluded from psychedelic research?
    - How should individuals with a history of psychotic disorders or suicidal ideation or behavior be considered in the context of psychedelic research?
25. Are there any ethical issues you've encountered in your own research with psychedelics that we haven't discussed yet?
    - How did you resolve them? Are they resolved?

Personal Experience

26. How has your experience studying psychedelics or observing study participants use psychedelics influenced your views about the risks and benefits of psychedelic research?

27. Have any personal experiences with psychedelics influenced your views about the risks and benefits of psychedelic research?
28. Have any of your personal or professional experiences related to psychedelics impacted your interactions with IRBs reviewing and overseeing your psychedelic research?

Resources

29. What specific resources or types of resources would be useful for IRBs reviewing psychedelic research?
30. If you could give IRBs responsible for psychedelic research one message, what would it be?
31. What specific resources would be useful in helping psychedelic investigators successfully navigate IRB review?
32. If you could give a researcher submitting their first psychedelics protocol to the IRB one piece of advice, what would it be?

Catchall

- Is there anything else important for IRB review of psychedelic research or psychedelic investigator engagement with IRBs that we haven't discussed yet?
